# Effect of suspension training on the balance ability of surfers without relying on vision

**DOI:** 10.3389/fphys.2025.1594228

**Published:** 2025-11-21

**Authors:** Zhaoyi Wang, Jiayang Zhou, Yong Ma, Zhihao Guo, Mengyao Jia, Weitao Zheng

**Affiliations:** 1 School of Intelligent Sports Engineering, Wuhan Sports University, Wuhan, China; 2 Key Laboratory of Sports Engineering of General Administration of Sports of China, Wuhan Sports University, Wuhan, China; 3 Specialised Research Centre for High-Quality Development of Competitive Sports, Wuhan Sports University, Wuhan, Hubei, China; 4 Engineering Research Center of Sports Health Intelligent Equipment of Hubei Province, Wuhan Sports University, Wuhan, China

**Keywords:** intervention training, dynamic balance, static balance, linear travel deviation test, eye-closed one-leg stand test

## Abstract

**Background:**

Surfing is an emerging Olympic sport that requires athletes to have excellent balance without relying on vision. This study introduces TRX (Total Resistance Exercise) suspension training into the balance training of surfing programs to investigate its effectiveness on the surfers’ balance ability without relying on vision.

**Methods:**

Thirty-two surfers from the Chinese National Surfing Team were randomly divided into a TRX group and a traditional balance training (TB) group, and the two groups were given intervention training for about 30 min three times a week for 8 weeks. Eye-closed one-leg stand and linear travel deviation tests were performed at different experiment stages to examine static and dynamic balance changes without visualization.

**Results:**

After intervention, both groups significantly improved left/right foot static balance (eyes-closed single-leg standing time) (F = 21.26 and 25.22, *p* < 0.001), with no significant inter-group or group × time interaction effects (all *p* > 0.05). For dynamic balance (linear travel deviation), both groups improved significantly with intervention (F = 23.41, *p* < 0.001), and inter-group difference was significant (F = 4.65, *p* = 0.039); the TRX group had smaller deviation than the TB group at E2 (5 weeks post-intervention, *p* = 0.021) and E3 (8 weeks post-intervention, *p* < 0.001), with a significant group × time interaction (F = 3.36, *p* < 0.05), showing better improvement in the middle-late intervention stage.

**Conclusion:**

TRX and TB were effectively able to improve surfers’ non-vision-dependent balance ability. However, TRX was more effective in improving dynamic balance in that situation. TB and TRX can be used to improve the static balance ability and dynamic balance ability for the first 5 weeks, and TRX can be applied to the balance training of surfers after 5 weeks.

## Introduction

Competitive surfing is an international professional water sport (Coyne et al., 2017) and is now listed for the 2020 and 2024 Summer Olympics (FernAndez-Gamboa et al., 2018). Competitive surfing success depends on the surfer’s ability to capture and ride the wave at its most critical part (i.e., closest to the break) while executing and completing innovative maneuvers (Mendez-Villanueva et al., 2006; Farley et al., 2012). For each surf, judges score the surf based on difficulty, main maneuver type, speed, power, and fluidity (Liu, 2015; Ferrier et al., 2018; Forsyth et al., 2021). This requires surfers to have great muscular endurance, explosive power (Lundgren et al., 2014; Anthony et al., 2016; Donaldson et al., 2022), excellent cardiorespiratory capacity (Bruton et al., 2013), and balance stabilization control (Farley et al., 2016a; Forsyth et al., 2017). Among them, balance is the foundation for surfers to perform surfing sports in the sea (Ma and Xu, 2020; Parsonage et al., 2017), which guarantees stabilizing the athlete’s body posture in the waves to perform various technical movements.

The perception systems for balance include the vestibular system (Liu et al., 2004), visual system (Ren et al., 2011), and proprioceptors (You and Wen, 2014). As surfing skill improves, athletes rely less on visual information to maintain upright posture (Chapman et al., 2008), which indicates an increased reliance on proprioceptive and vestibular sources of information (Paillard et al., 2011). Once surfers begin to develop a high level of expertise and athletic experience, the inner demands of surfing may become less reliant on visual input (Paillard et al., 2011), and proprioceptive stimulation may dominate, thereby maintaining limb balance stability (Dowse et al., 2021). Long-term participation in recreational surfing leads to specific neuromuscular adaptations that control muscle force production and posture, where posture control is more dependent on proprioception (eyes closed) (Frank et al., 2009). Repeated experience in elite surfers may enhance balance through neural adaptations that are less dependent on visual inputs, thus reducing the need for visual contributions to postural control (Hrysomallis, 2011). Therefore, without relying on vision, surfers must also have a strong ability to control body balance. At present, research on surfing programs worldwide has mostly focused on analyzing the scoring rate of surfing skill movements (Donaldson et al., 2022; Farley et al., 2016b), competition results (Forsyth et al., 2021; Ma et al., 2023), and the surfing physical fitness training (Ma and Xu, 2020; Parsonage et al., 2020), while only few studies have explored how to better train balance in conjunction with the athletic characteristics of surfing.

Total Resistance Exercise (TRX), as a specific type of suspension training, emphasizes using one’s own body weight as resistance. It aims to suspend a certain body part using a rope, imposing an unstable state by stimulating the proprioceptors to strengthen the control and regulation of muscle tension gradually (Li et al., 2008). TRX training ultimately enhances the neuromuscular system harmonization effect (Campa et al., 2018; Demirar et al., 2021) and thus effectively improves joint stabilization and neuromuscular control (Aguilera-Castells et al., 2019). Hence, applying it to athletic training can enhance body balance and control (Seiler et al., 2006), making it easier for athletes to perform difficult movements. TRX is widely applied in athletic training in various sports, such as improving the balance stabilization of athletes in track and field (Qiao and Yuan, 2010), taekwondo (Dong et al., 2019), tennis (Fu and Li, 2014) and physical control (Demirar et al., 2021; Wang et al., 2016), enhancing swimmers’ balance (Wang et al., 2012), improving balance stability and special techniques of athletes in skill-based events (Li et al., 2010), and enhancing the movement control of soccer players in unstable states (Chen and Sun, 2014). There is currently little research on TRX’s effectiveness in the surfers’ balance ability without relying on vision.

Because TRX stimulates proprioception in unstable environments, it in turn improves the stabilizing ability to control body balance. Therefore, it can effectively strengthen the proprioceptive ability after mitigating the visual input, which is suitable for the balance training of excellent surfers. In contrast, traditional balance training is usually performed through a stable support surface, with the main purpose of improving body stability and control (Qiao and Yuan, 2010), but this method has limitations in activating and strengthening the core muscles and other supporting muscles, making it difficult to comprehensively improve balance, coordination and strength.

Currently, it has been found by the coaches of the Chinese National Surfing Team that the effects of traditional balance training methods have stabilized and exhibit limited efficacy in further enhancing athletes’ performance, while the effects of TRX Suspension Training in surfing training remain unclear. Therefore, a study has been initiated to explore more suitable training methods for surfing, so as to more effectively enhance athletes’ balance ability. This study hypothesizes that TRX can significantly improve surfers’ balance ability without relying on vision. At the same time, the differences in the effects of TRX and Traditional Balance training (TB) on surfers’ balance ability were compared. A more suitable balance training method for surfers was explored with the aim of helping surfers to improve their athletic performance.

## Materials and methods

### Participants

The predicted sample size was calculated using G Power software version 3.1.9.2. (Heinrich-Heine-Universität Düsseldorf, Düsseldorf, Germany). Correlation analyses using a medium effect size value of 0.5 required a minimum of 26 subjects at α = 0.05 and a statistical power of 0.80. Considering the risk of uncertainty, all athletes from the China National Surfing Team were enrolled in the intervention experiment, and all participants were screened for qualification. Inclusion criteria were as follows: before the experiment, all athletes were physically examined to determine the absence of sports injuries, diseases, and normal motor function; athletic achievements: individuals ranked in the top 12 nationally, teams ranked in the top 8 nationally (including National Games, National Championships, etc.).

Participants are at the same stage of the sports season, which can avoid training variables that may influence the results when participants are in different phases of the season. The exclusion criteria were as follows: knee and ankle arthrodesis, fractures, history of previous surgery elsewhere in the knee, and other forms of muscular or skeletal injuries to the lower extremity; prior injury to the lower extremity of at least 3 months and the presence of pathology that may impair balance. After screening, 36 qualified Chinese Surfing Team athletes were selected to participate in the study. During the 8-week intervention period, 4 athletes missed part of the training due to personal reasons, leading to a final sample size of 32 athletes to be included in the statistical analysis as participants in this study, including 19 national elite athletes and 13 first-class athletes. National elite athletes represent the highest competitive level in Chinese surfing, comparable to international standards, while first-class athletes are also highly skilled and recognized within the national sports system.

This study utilized a single-blind design, in which participants were unaware of which group they were assigned to during the experiment, so as to prevent the influence of participants’ expectations or psychological factors on the experimental results. Meanwhile, to ensure the balance of sex, age, height, weight, and other baseline characteristics between the two groups, this study adopted a block randomization method: first, all participants were divided into several blocks based on age, gender, and exercise level; then, within each block, participants were further randomly assigned to the two experimental groups using a computer-generated random number program, ensuring the comparability of baseline characteristics between groups. In addition, the grouping process, measurement operations, and blinding implementation of this study were each independently completed by different fixed researchers; all these researchers were unaware of the participants’ group assignments during the experiment to avoid measurement bias and the interference of subjective factors.

**FIGURE 1:**
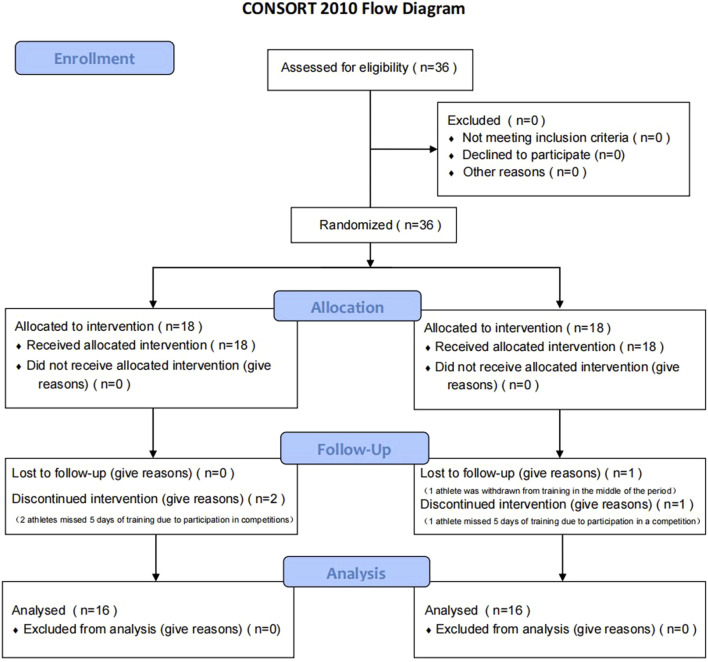
The Consort flow diagram.

Additionally, all grouping results and participant allocations were recorded uniformly after the experiment and only unblinded by designated personnel during the data analysis stage, further enhancing the credibility and transparency of the study. This rigorous randomization and blinding process, combined with balanced control of key baseline characteristics, effectively improved the scientific validity of the experimental design and the accuracy of the results. Finally, athletes were randomly divided into TRX (TRX suspension training) group (n = 16) and TB (Traditional Balance training) group (n = 16). Table 1 shows the basic characteristics of the participants after grouping, and Figure 1 illustrates the Consort flow diagram.

**TABLE 1:** Basic information of subjects (sex, number, age, weight, height and years of training).

Group	Gender & numbers	Age/y	Height/cm	Weight/kg	Training years/y
TRX	8M and 8F	15.38 ± 2.15	164.69 ± 7.78	52.81 ± 7.64	2.88 ± 0.48
TB	8M and 8F	15.44 ± 2.09	164.69 ± 7.24	52.31 ± 8.08	2.88 ± 0.70
*p*	--	0.936	1.000	0.863	1.000

Height and weight collection equipment for height and weight tester (Kefun Group, Model: KF-1328, Zhejiang, China). Measuring range: 0.1–180 KG, 70-190CM. TRX, TRX, suspension training group; TB, Traditional Balance training group. M, Males. F, females.

All subjects were informed and agreed to participate in all experiments of this study by signing an informed consent form. Meanwhile, the participants provided informed consent to publish the images in the study. This study confirmed that all methods were performed in accordance with relevant guidelines and regulations, and the study was approved by the Ethics Committee of Wuhan Sports University (No: 2021030).

### Study procedure

#### Intervention design

This study was conducted during the 2022 National Surfing Team training period, and all the training, diet and work/rest of the athletes were done in a unified and standardized way. The experiment took place in the physical training room of the China National Surfing Team in Lingshui Autonomous County, Hainan Province, China. Specifically, all training activities, including fitness routines and surfing practice, were centrally managed and scheduled by the national team to ensure consistency and minimize potential confounding variables. This centralized approach allowed to maintain uniformity across all aspects of the participants’ routines, with the only variation being the balance training interventions applied to the two study groups.

The study adopted a “1 + 8” experimental period arrangement, with 1 week of adaptive training and 8 weeks of formal experiments. The adaptive training week was designed to enable the athletes to complete the training movements in a more standardized way to provide good preparation for the accuracy and standardization of the formal experiments.

The formal experiment was conducted for 8 weeks of intervention training (Wei et al., 2022), three times a week for about 30 min each time. The training movements in both groups were based on balance and core training. Meanwhile, the acceptance of training movements and the athletes’ training effect were monitored throughout this phase. Besides, the formal experiment’s training load was formulated considering the optimal intensity (number of times or duration) of the standardized movements each individual could achieve during the training. The training intensity was adjusted according to the actual state of the athletes, ensuring that the training loads of the two groups were the same and that the loads as a whole obeyed the principle of gradual progression (Wang et al., 2016). Specifically, training intensities were scheduled based on the optimal intensity of the standardized movements that each athlete demonstrated during the adaptive training period, either in terms of the number of times the movement was completed or the duration. By observing the performance of the athletes, the research team set an initial training intensity for each athlete that would challenge athlete current abilities without causing excessive fatigue. Throughout the 8-week formal experiment, the training intensity followed a gradual incremental progression to continuously stimulate the athlete’s body and promote adaptation and improvement. The actual condition of the athletes was closely monitored, and if fatigue or other discomforts occurred, the training intensity was adjusted accordingly to prevent injuries and maintain the sustainability of the training. In order to ensure the quality and consistency of the training program, each group was supervised and guided by a national team physical fitness coach, which further ensured the scientific and effective training.

### Intervention content

The 8 weeks training was divided into 3 phases: Weeks 1-2 of basic adaptation phase (E1), Weeks 3-5 of quality enhancement phase (E2), and Weeks 6-8 of consolidation and strengthening phase (E3), with the intensity and difficulty of the training gradually increasing (Fu and Li, 2014). During the entire intervention training, the athletes were only allowed to perform the movements required, with no other balance movement training.

The two groups in the E1 phase were mainly based on static training movements to allow the athletes to adapt to the initial training rhythm (Wang et al., 2024). In the E2 phase, the two groups combined static and dynamic training, and the load and difficulty of the training increased. Dynamic training movements were executed in both groups in the E3 phase, and the difficulty and load were again increased. The details of the training program are shown in Table 2.

**TABLE 2:** Training content during the 8 weeks of intervention in both groups of athletes.

Training phase	Groups	Training movement	Number of groups	Time/Reps	Group interval
E1	TRX	Suspended prone planks, suspended legs supine hip bridge, suspended lateral one-handed brace, suspended plank balance	4	20–40s (each side)	60s
	TB	Supine leg raises, plank support, hip bridge, lateral bridge	4	20–40s (each side)	60s
E2	TRX	Suspended prone legs supported open and close, forward and backward movement of the suspension plate support, suspended single leg V-support, suspended prone support group, suspended straight leg flexion	4	30–50s/12-16reps (each side)	60s
	TB	Plate support movement, knee rolls, straight legged foot rolls, lateral rolls, air cycling	4	30–50s/12-16reps (each side)	60s
E3	TRX	Suspended single-legged double-armed support prone leg tucks, suspended single-legged one-handed lateral support leg suction, suspended single-legged prone support lateral knee lifts, suspended prone support knee lift runs, suspended prone one-handed rotations	4	40–60s/16-50reps (each side)	60s
	TB	Supine chin ups, single arm support turn, one-handed prone knee lifts, group body movements, prone mountain running	4	40–60s/16-50reps (each side)	60s

In this study, the training programs of both groups were decided through in-depth discussion and research by a group of experts consisting of outstanding coaches, physical trainers and other experts, to ensure that the training methods were not only scientific and effective, but also fully in line with the special requirements of surfing techniques. Specifically, the training movements and programs of the TRX group were formulated by the expert group by combining the characteristics of surfing, referring to the experience of other similar sports (e.g., skateboarding, freestyle skiing, etc.), and synthesizing the successful application cases of TRX suspension training in other sports. In contrast, the training program for the TB group was directly adopted from the original balance training program used by the Chinese National Surfing Team. This program has been implemented in the surfing team for some time and can effectively evaluate the advantages and disadvantages of TRX suspension training in improving balance ability. Through this control design, this study can not only verify the innovation and applicability of the TRX training method, but also provide a more scientific and efficient practical reference for the physical training of surfing.

### Test indicators

All tests were conducted in the same location (Lingshui County, Hainan Province, National Surfing Team Physical Training Room), in the same environment (temperature 20°–27°, humidity 68%–78%, quiet indoors), and during the same period of time (7:00–10:00 p.m.). In order to avoid subject interaction, the tests were administered on a one-by-one entry basis, with the previous person completing the test before the next person could enter. Two testers were responsible for each evaluation indicator, and the assignment of tasks was consistent throughout the study period to ensure data accuracy and reliability. The tests were divided into four sessions according to training time (Table 3).

**FIGURE 2:**
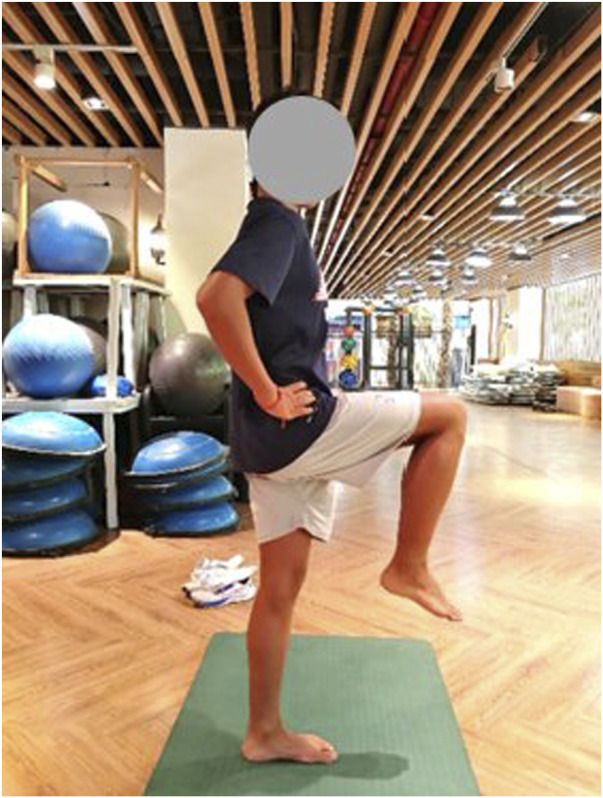
Eye closed one leg stand test chart.

**FIGURE 3:**
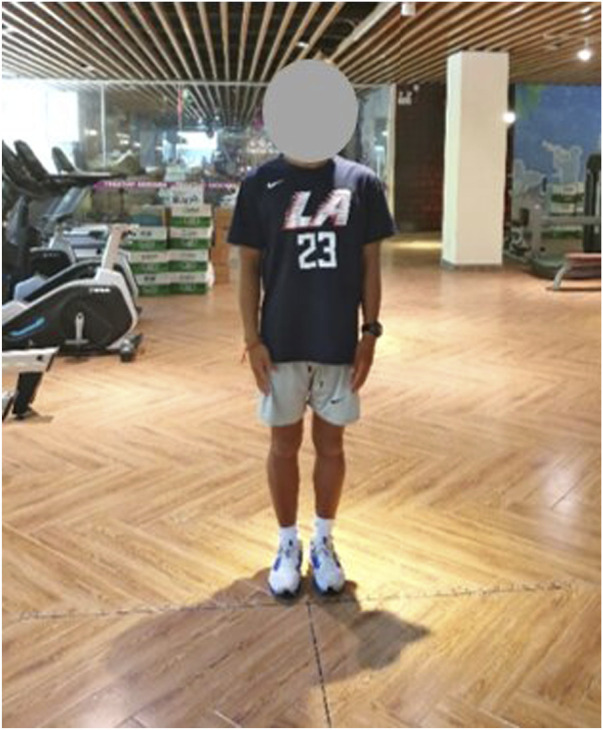
Linear travel deviation test chart.

1. Eye-closed one-leg stand test: The test relies on the balance receptors in the vestibular organs of the brain and the coordinated movement of muscles from the whole body when the participant closes his or her eyes (Li, 2022; Yuan et al., 2013). Static balance was evaluated by the amount of time the participant maintained at his body’s center of gravity on a one-legged support surface, keeping standing on one leg for as long as possible (Figure 2). Each participant’s leg was tested twice at 2-min intervals during the test, and the final result was taken as the best score for each leg. The best score was selected because static balance in surfing is reflected in the maximum ability to maintain a stable posture (e.g., balance duration after standing up), and the best score better reflects the true potential of static balance.
2. Linear travel deviation test: tests the participants’ dynamic balance, vestibular perception, and sense of spatial orientation without relying on vision (Cao, 2021). The test was performed with the participant’s eyes closed, and after clarifying the starting and stopping points, the participant moved forward and backward for 10m, respectively (Figure 3). We measured the deviation distance of the final landing point from the start and stop points, and the test takes the average of the two deviation distances as the result. The smaller the deviation distance, the better the participant’s dynamic balance. Each participant was tested twice, and the final result was the best of the two tests. The average was selected because dynamic balance in surfing is reflected in the continuous stability to respond to waves (e.g., spatial orientation during paddling/gliding); the average reduces interference from instantaneous status and better aligns with practical needs.

**TABLE 3:** Organization of the four tests.

Organization of the training phase	Testing time
E0	Before the experiment starts
E1	Test after completion of the basic adaptation phase (Weeks 1–2)
E2	Test after completion of the quality enhancement phase (Weeks 3–5)
E3	Test after completion of the consolidation and strengthening phase (Weeks 6–8)

### Statistical analyses

Data were analyzed using SPSS Statisticel 26 software (Version 26.0, IBM Corporation, Armonk, New York, United States) and expressed as mean ± standard deviation (X ± SD). First, homogeneity tests were performed on the basic information and balance ability of participants in the two groups to ensure the rationality of random grouping and that the initial balance ability of participants was at the same level. For the balance ability indicators before and after intervention, Two-way Repeated Measures ANOVA was used for analysis, where the independent variables were group (TRX group, TB group) and time (E0, E1, E2, E3), and the dependent variables were the balance indicators. When a significant group main effect, time main effect, or group × time interaction effect was detected, Bonferroni correction was applied for *post hoc* tests to identify specific differences between time points within groups and between groups. Partial eta-squared ( $\eta_{p}^{2}$ ) was also reported to quantify the practical significance of effects, with the following criteria: $\eta_{p}^{2}$ = 0.01 for a small effect, $\eta_{p}^{2}$ = 0.06 for a medium effect, and $\eta_{p}^{2}$ = 0.14 for a large effect. In statistical tests, *p* > 0.05 indicated no significant difference, *p* < 0.05 indicated a statistically significant difference, and *p* < 0.01 indicated a highly statistically significant difference.

## Results

### Homogeneity test

In order to be capable of knowing whether the athletes were at the same level of basic information and balance before the experiment, the relevant indexes of the two groups of athletes were tested. The results showed there was no significant difference (p > 0.05) between the two groups of participants in terms of physical characteristics (Table 1) and static and dynamic balance ability without relying on vision (Table 4), which were comparable.

**TABLE 4:** Comparison of balance ability between the two groups of athletes before the experiment.

Balance ability	Test indicator	TRX	TB	t	*p*
Static balance	Eyes closed left foot stand (s)	57.75 ± 38.61	58.01 ± 32.38	−0.190	0.985
	Eyes closed right foot stand (s)	47.50 ± 34.21	47.94 ± 27.23	0.039	0.969
Dynamic balance	Linear travel deviation test (cm)	69.27 ± 22.77	68.67 ± 23.94	0.070	0.945

### Changes in static balance before and after the intervention

Table 5 presents the changes in static balance of the left and right feet before and after the intervention. For the static balance of the left foot, the F - value for the group effect was 1.17, with the *p* of 0.288, and the $\eta_{p}^{2}$ was 0.038, indicating no significant difference in the overall level of left - foot static balance ability between the TRX group and the TB group. The F - value for the time effect was 21.26, with the *p* less than 0.001, and the $\eta_{p}^{2}$ was 0.414, suggesting that the left - foot static balance ability of both groups improved significantly over time. Post - hoc tests with Bonferroni correction showed significant differences in left - foot balance ability at each time point (from E0 to E1, E1 to E2, and E2 to E3) in both the TRX group and the TB group (all *p* < 0.01). The F - value for the group × time interaction effect was 0.75, with the *p* of 0.526, and the $\eta_{p}^{2}$ was 0.024, meaning that there was no significant difference in the change trends of left - foot static balance.

**TABLE 5:** Results of the eye closed one leg stand test in both groups.

Indicator	ANOVA effects	F - value	*p*	$\eta_{p}^{2}$	Post - hoc results (within - or between - group comparisons, *p*)
Eyes closed left foot stand (s)	Group effect (group)	1.17	0.288	0.038	Between - group comparison: No significant difference (*p* > 0.05)
	Time effect (time)	21.26	<0.001	0.414	Within - group comparison: For the TRX group, E0 < E1 < E2 < E3 (all *p* < 0.01); for the TB group, E0 < E1 < E2 < E3 (all *p* < 0.01)
	Group × time interaction effect	0.75	0.526	0.024	No significant interaction effect *(p* > 0.05)
Eyes closed right foot stand (s)	Group effect (group)	2.52	0.123	0.077	Between - group comparison: No significant difference (*p* > 0.05)
	Time effect (time)	25.22	<0.001	0.457	Within - group comparison: For the TRX group, E0 < E1 < E2 < E3 (all *p* < 0.01); for the TB group, E0 < E1 < E2 < E3 (all *p* < 0.01)
	Group × time interaction effect	1.33	0.272	0.043	No significant interaction effect *(p* > 0.05)

Regarding the static balance of the right foot, the F - value for the group effect was 2.52, with the *p* of 0.123, and the $\eta_{p}^{2}$ was 0.077, showing no significant difference in the overall level of right - foot static balance ability between the two groups. The F - value for the time effect was 25.22, with the *p* less than 0.001, and the $\eta_{p}^{2}$ was 0.457, indicating that the right - foot static balance ability of both groups improved significantly over time. Post - hoc tests with Bonferroni correction indicated significant differences in right - foot balance ability at each time point (from E0 to E1, E1 to E2, and E2 to E3) in both the TRX group and the TB group (all *p* < 0.01). The F - value for the group × time interaction effect was 1.33, with the *p* of 0.272, and the $\eta_{p}^{2}$ was 0.043, suggesting that there was no significant difference in the change trends of right - foot static balance ability between the two groups at different time points.

### Changes in dynamic balance before and after the intervention

Table 6 presents changes in dynamic balance (linear travel deviation distance, unit: cm) of both groups before and after the intervention. Group effect analysis showed an F-value of 4.65 (*p* = 0.039) with the $\eta_{p}^{2}$ of 0.134, indicating a significant difference in overall dynamic balance ability between the two groups. Post-hoc tests with Bonferroni correction revealed: at E2 (5 weeks post-intervention) and E3 (8 weeks post-intervention), the TRX group had significantly smaller deviations than the TB group (*p* = 0.021 < 0.001); no significant difference was observed at E0 (pre-intervention) and E1 (2 weeks post-intervention) (both *p* > 0.05).

**TABLE 6:** Comparison of the results of two groups of linear travel deviation tests at different phases.

ANOVA effects	F - value	*p*	$\eta_{p}^{2}$	Post - hoc results (within - or between - group comparisons, *p*)
Group effect (group)	4.65	0.039	0.134	Between - group comparison: TRX < TB at E2 (*p* = 0.021), TRX < TB at E3 (*p* < 0.001); No difference at E0 and E1
Time effect (time)	23.41	<0.001	0.438	Within - group comparison - For the TRX group, E0 > E1 > E2 > E3 (all *p* < 0.001) - For the TB group, E0 > E1 > E2 (p < 0.01), and E2≈E3 (*p* > 0.05)
Group × time interaction effect	3.36	<0.05	0.101	Significant interaction effect: The improvement amplitude of the TRX group after E2 is greater than that of the TB group (*p* < 0.01)

Time effect analysis showed an F-value of 23.41 (*p* < 0.001) with the $\eta_{p}^{2}$ of 0.438, demonstrating that both groups’ dynamic balance ability improved significantly with prolonged intervention. Within-group comparisons: the TRX group showed a stepwise decrease in deviation (E0>E1>E2>E3) with significant differences between adjacent time points (all *p* < 0.001); the TB group had a significant decrease in deviation from E0 to E2 (E0>E1>E2, *p* < 0.01) but no significant change from E2 to E3 (*p* > 0.05).

Group × time interaction effect analysis showed an F-value of 3.36 (*p* < 0.05) with the $\eta_{p}^{2}$ of 0.101, indicating a significant interaction in change trends between the two groups. Specifically, after E2, the TRX group had a significantly greater decrease in deviation than the TB group (*p* < 0.01), meaning TRX training had a better improvement effect in the middle and late stages of intervention.

## Discussion

### Analysis of changes in the static balance

The results of the TRX group showed that TRX improved the athlete’s ability to stand on one foot with eyes closed, i.e., static balance without relying on vision. This is because TRX trains the body to perform movements in an unstable state, and the method exercises the athlete’s ability to control the body in this state (Wei et al., 2009). The body constantly moves from imbalance to balance during the training (Zheng and Qu, 2022), and this pattern of movement can adequately stimulate one’s vestibular system (Wang et al., 2012) to achieve the optimal evoked effect on proprioceptive organs (Qiao and Yuan, 2010), improving the neuromuscular control of surfers.

While in the test, the body is unstable because of the reduction of the body support area. At this time, the central nervous system must send commands to the lower limb muscles to ensure body balance (Zhang et al., 2012). Therefore, by exercising the ability to control the body in unstable conditions, the results of closed-eye single-leg standing were also significantly improved, suggesting that TRX can be a good way to improve the static balance of surfers when they do not rely on vision. In contrast, the traditional means of lumbar-abdominal balance training provides continuous stimulation to the muscles of a particular body part (Wang et al., 2012), aiming to improve the strength capacity of the abdomen, lower back, and lower limb joints (Seiler et al., 2006). For example, the side curls, aerial cycling, and prone mountaineering run in the TB group effectively worked on abdominal and lower extremity strength stabilization, so the improvements in the test results were all more obvious.

Both TRX and TB groups showed significant variability in changes in static balance before and after the intervention, indicating that both methods can improve the surfers’ static balance without relying on vision. Comparing the training effects achieved by the two methods, although the test scores of the TRX group were higher than those of the TB group, there was no significant difference in the results. This finding is more controversial at the current time, other researchers have compared the static balance of surfers, Chapman et al. (2008) assessed the static balance of elite and intermediate surfers on a balancing platform and found no difference between the two groups (Chapman et al., 2008). Furthermore, Alcantara et al. (2012) found no differences in static balance performance between recreational surfers and controls (Alcantara et al., 2012). However, as surfing takes place in an ever-changing and volatile environment, static equilibrium enhancements and needs may not be very clearly discernible (Hrysomallis, 2011).

The above findings are consistent with the findings of the present experiment, but equally studies have shown that TRX training is superior to traditional training (Cao, 2021; Sun et al., 2012). For the current experiment, the reason for the lack of significant difference is that the TRX training in this study was more focused on developing the surfers’ body control from stretching to massaging and back again, i.e., control of the trunk. This is because the foundation of any movement a surfer makes during surfing requires the athlete to go from paddling to taking off and riding before completing other technical movements. The trajectory of the human body during this phase is from a plank movement in the prone position to a supported rise to standing on the plank (Anthony et al., 2016). Regarding movement selection, this study focused on the strength and control of the core region of the upper limb, such as suspended straight leg flexion and suspended plate support for anterior and posterior movement. However, the eye-closed one-leg stand test is more biased toward the requirements of lower limb strength and knee and ankle stability in this aspect of muscle control, such as the hanging single-leg prone adductor leg exercise and the hanging single-leg reverse lunge exercise (Cao, 2021). Therefore, the focus of the intervention content varies, leading to differences in the outcome.

Meanwhile, in the arrangement of intervention phases, this study in the first phase of training focused on controlling the trunk under static training, and improving joint stability and muscle strength was not particularly significant. The latter two training phases began to combine the training of lower limb strength and joint flexibility in the dynamic mode. Therefore, the training for lower limb joint flexibility and muscle strength under static conditions is on the low side, while the eye-closed one-leg stand test belongs to the holistic test under static conditions (Yuan et al., 2013). Thus, there is no significant difference between the effects of the two training methods on a closed one-leg stand.

### Analysis of changes in dynamic balance

This study reveals that some of the athletes in the TRX group measured excellent scores with deviation distances within 10 cm at the E2 test phase, and there were even individual athletes who measured perfect scores with deviation distances of 0 at that test phase. Thus, this study concludes that this evaluation indicator has limitations for evaluating balance ability after achieving a perfect score. Therefore, to a certain extent, this test method cannot be used to determine the athletes’ dynamic balance ability without relying on vision in the long term. At the same time, for certain surfers, 5 weeks of TRX can improve their dynamic balance without relying on vision. In contrast, although the results showed that TB was equally effective in improving surfers’ ability, the TB group athletes did not have perfect deviation scores (0 cm deviation) or close to perfect.

Therefore, the test limits did not affect the TB group, which argues for the advantages of TRX over TB. Due to the ever-changing environments and high level of instability required for surfing, surfers must develop a number of neuromuscular skills (agility, balance, muscle strength and flexibility) for better performance. This involves visual deprivation (eyes opening or closing) and impairment of somatic sensation (stabilizing surfaces or using bubbles) (Alcantara et al., 2012). The training methods in this study created an unstable environment, trained somatosensory perception, and enhanced dynamic balance. The main influences on dynamic balance are the ability to control body posture and ankle muscle strength (Peng and Li, 2020). The TRX group arranges dynamic movement training in the quality enhancement and consolidation phase, and it aims to strengthen the stabilizing ability of the trunk, the control ability, and the flexibility of the joints when the athlete is in an unstable state so that it can effectively improve the dynamic balance ability of surfers.

A comparison of the differences in the effects of the two training methods reveals that the differences appeared after 5 weeks of training and became more and more significant as the length of training accumulated. This is because TB lacks the exercise of proprioception, the vestibular system, ankle stability, and lower extremity muscle strength. Traditional balance training typically relies on static, stable surfaces and often focuses on isolating specific muscle groups and providing continuous stimulation to that area with minimal involvement of other muscle groups and a low level of coordinated training between muscles. In contrast, TRX is inherently more challenging in terms of physical stability and inter-muscular coordination as it involves a more dynamic and unpredictable environment. During the initial phase of training, both groups may have made progress in building foundational balance and core strength, albeit at potentially different rates. However, by week 5, participants in the suspension training group may have reached a higher level of adaptation to unstable conditions, which is critical for surfing performance. TRX strengthens the stabilizing power of the rectus abdominis, internal and external abdominal obliques, transversus abdominis, and pelvic floor muscles through movement control, stimulates the vestibular system of the body (Xiao et al., 2001), and activates the tension receptors and pontine reticular spinal cord pathways to improve the athlete’s proprioceptive abilities (Wang et al., 2016). Meanwhile, TRX can effectively activate local stabilizing muscles and overall prime movers to improve intramuscular coordination (Sun et al., 2010). For instance, TRX can effectively improve muscle coordination between abdominal and back muscles, quadriceps and hamstrings, tibialis anterior, and calf triceps in controlling trunk stabilization (Li, 2022; Wang et al., 2025), whereas it is difficult to achieve the effect of multi-muscle group force generation with the flexion and extension exercises of the traditional training (Niu and Qiao, 2018). Therefore, TRX is more effective in improving athletes’ motor neuron control, muscle proprioceptive abilities, and vestibular system modulation.

During the experiment, the athletes in the TRX group often felt a burning sensation in the muscle groups of the core area after training for a movement and felt that they needed to apply full body force to control their bodies. In addition, some seemingly unrelated to the action of the muscle groups will also appear in different degrees of fatigue and soreness of the response. Hence, some traditional training methods cannot be practiced through this form of exercise, significantly improving the performance of the TRX group compared to the TB group. The results indicated that TRX improves surfers’ dynamic balance without relying on vision compared to TB. The findings of this indicator demonstrate consistency with the research results of Cao (Cao, 2021), indicating that the data of the linear travel deviation test in this study are real and valid.

The limitations of this study include the surfers’ balance ability was analyzed as a whole, and no comparative study was done on the differences between the left and right leg results, the lack of correlation analysis between athlete performance before and after training, the lack of the chronic effects of the group differences, and the lack of exploration of the impact of different methods on gender differences among surfers. In the future, further research can be conducted on the differences between the left and right leg, the correlation between training methods and sports performance, whether the observed results persist after the training without vision as well as the differences in the effectiveness of training methods on sports training for different genders. In addition, the linear travel deviation test used in this study has limitations in evaluating balance ability without relying on vision. This indicator may not be able to fully reflect the athletes’ balance performance in different environments and conditions, especially when tracking training effects over time. Future research should explore more comprehensive and sensitive methods of balance assessment to more accurately measure the long-term effects of training interventions on athletes’ balance ability, specifically by incorporating biomechanical tools such as force platforms and balance meters to accurately collect key indicators, quantify subtle changes in balance control, and improve assessment scientificity.

## Conclusion

Both TRX and TB can effectively improve surfers’ balance ability without relying on vision. Although there was no significant difference between the two in static balance improvement, TRX was more effective than TB in improving dynamic balance, and the advantage persisted with increasing training time. Therefore, both TB and TRX are suitable for static balance training, while TRX is more recommended for dynamic balance training. It is recommended to choose the appropriate method according to the training goal.

## Data Availability

The original contributions presented in the study are included in the article/Supplementary Material, further inquiries can be directed to the corresponding authors.
