# Supplementary material for: Effect of suspension training on the balance ability of surfers without relying on vision
Source: Front Physiol. 2025 Nov 21;16:1594228. doi: 10.3389/fphys.2025.1594228 (PMC12678109; doi:10.3389/fphys.2025.1594228)
Supplement: Supplementary file 1 [file DataSheet1.pdf]

Effect of Suspension Training on the Balance Ability of Surfers without Relying on Vision

Method

(i). Participants:

36 professional surfers who are currently members of the Chinese National Surfing Team were randomized equally into the TRX and TB groups to receive an 8-week balance intervention.

(ii). Intervention content:

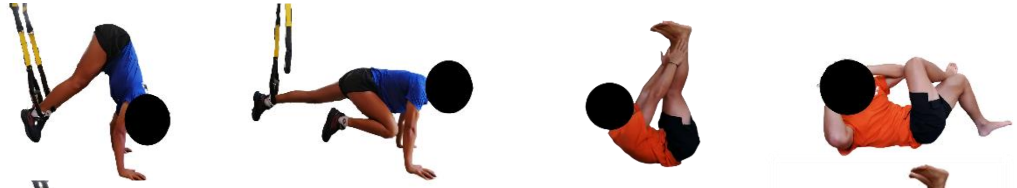

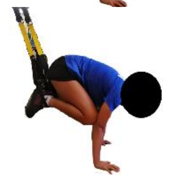

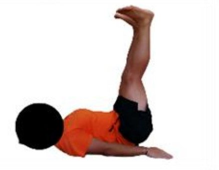

- 30 minutes 3 times a week for 8 weeks.
  - 1-2 weeks: basic adaptation phase
  - 3-5 weeks: quality enhancement phase
  - 6-8 weeks: consolidation and strengthening phase

(iii). Test indicators

◆ Eye Closed One Leg Stand Test ◆ Linear Travel Deviation Test

✓ The Paired Samples t-test for within-group comparisons

✓ The Independent Samples t-test for between-group comparisons

Note: **Abbreviation** list is covered in manuscript

Results

◆ Eye Closed One Leg Stand Test

TRX

E1 phase:
left foot support ↑↑
right foot support ↑↑

E2 phase:
left foot support -
right foot support -

E3 phase:
left foot support ↑
right foot support ↑

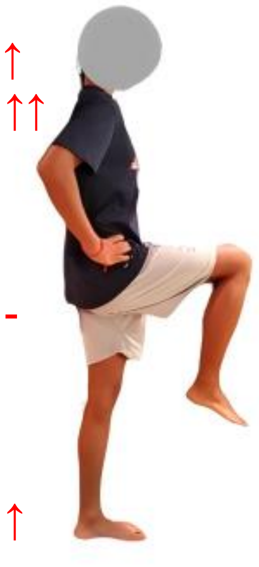

TB

E1 phase:
Left ↑↑
right ↑↑

E2 phase:
left -
right -

E3 phase:
left -
right ↑

◆ Linear Travel Deviation Test

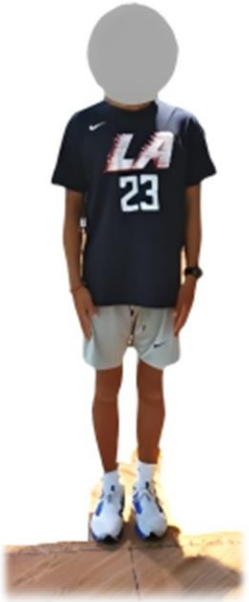

E1 phase:
TRX deviation ↓↓
TB deviation ↓↓

E2 phase:
TRX deviation ↓↓
TB deviation ↓↓

E3 phase:
TRX deviation ↓↓
TB deviation ↓↓

Differences in the effectiveness of the two training methods: no significant difference in eye-closed one leg stand, while the linear travel deviation TRX group was significantly better than the TB group from 5 weeks of training until the end of the intervention.

Conclusions

Both TRX and TB can effectively improve surfers' balance ability without relying on vision. Although there was no significant difference between the two in static balance improvement, TRX was more effective than TB in improving dynamic balance, and the advantage persisted with increasing training time. Therefore, both TB and TRX are suitable for static balance training, while TRX is more recommended for dynamic balance training. It is recommended to choose the appropriate method according to the training goal.
